# Case of Cardiac Arrest Treated with Extra-Corporeal Life Support after MDMA Intoxication

**DOI:** 10.1155/2019/7825915

**Published:** 2019-03-07

**Authors:** Pierre Voizeux, Romain Lewandowski, Theresa Daily, Omar Ellouze, Olivier Bouchot, Belaïd Bouhemad, Pierre-Grégoire Guinot

**Affiliations:** ^1^Department of Anaesthesia, Dijon University Hospital and University of Dijon, Dijon, France; ^2^Department of Cardiovascular Surgery and University of Dijon, Dijon University Hospital, Dijon, France

## Abstract

**Objective:**

To describe the case of a patient who developed a serotonin syndrome due to a 3,4-methylenedioxymethamphetamine ingestion with electrical storm and refractory cardiac arrest.

**Design:**

Case report.

**Study Selection:**

ICU of a university hospital.

**Patient:**

A 22-year-old man transferred to the emergency room with hyperthermia, tremors, and mydriasis presented a cardiac arrest due to ventricular fibrillation.

**Interventions:**

We implemented extra-corporeal life support combined with vasoactive drugs. Later, he also benefited from renal replacement therapy and mechanical ventilation.

**Measurements and Main Results:**

We were able to rapidly regulate our patient's temperature and we weaned all hemodynamic support in the first week of hospitalisation.

**Conclusion:**

Extracorporeal life support has several advantages as part of the management of hemodynamic instability induced by serotonin syndrome.

## 1. Introduction

Arrhythmia is one of the main causes of cardiac arrest among young patients. We report the case of a 22-year-old male with a past history of toxic abuse presenting with hyperthermia and severe arrhythmia who was found to have a serotonin syndrome induced by a 3,4-methylenedioxymethamphetamine (MDMA) ingestion. Many risk factors of serotonin syndrome are identified in the literature and MDMA abuse is one of them [1–3]. However, this is the first case describing a successful recovery from a refractory cardiac arrest secondary to a MDMA serotonin syndrome treated with extra-corporal life support (ECLS).

## 2. Case Report

Firemen found a 22-year-old male in his car presenting hyperthermia, bilateral mydriasis, and general contracture. The patient was brought to the emergency room of our teaching hospital in Dijon. According to his family, his medical history only included a polydrug use of cannabis, cocaine, and MDMA. He had no family medical history. A CT-scan was performed upon arrival and revealed no lesions. Toxic analysis returned positive for MDMA and cannabis.

Initial physical examination showed a patient with a Glasgow Coma Scale of 13/15, mydriasis, sweating, and a body temperature of 37.1°C. He had no tremors or any clinical signs of a pyramidal syndrome. His respiratory rate was 26/min, his heart rate 72 bpm, and his blood pressure 167/110 mmHg. The patient then quickly became comatose and presented hyperthermia (42.5°C), tachycardia (172 bpm), and high blood pressure (175/101 mmHg). The first arterial blood gas displayed acidosis with a pH at 7.238 and severe hypoxemia with a PaO_2_ at 99 mmHg on 100% O_2_. The bloodwork also showed HCO_3_^−^ 20.4 mmol/L, sodium 134 mmol/L, potassium 4.6 mmol/L, creatinine 118 *μ*mol/L, uraemia 5.4 mmol/L, and CPK 1197 mmol/L.

He was therefore transferred to our ICU where he was sedated, curarized, and intubated, and four litres of crystalloid were infused. A few minutes later, he suffered from ventricular tachycardia then ventricular fibrillation followed by electromechanical dissociation. He received an external electric shock as well as intravenous dantrolene. Because of sustained hemodynamic instability without rhythmic recovery, extra-corporal life support was implanted with introduction of norepinephrine and dobutamine.

The heart function rapidly improved after a few episodes of ventricular tachycardia that were treated with intravenous amiodarone. The patient was supported with norepinephrine for the first day and with dobutamine until the 4^th^ day when a transthoracic echocardiography revealed a LVEF of 35% with normal cardiac output. The ECLS was withdrawn on the 4^th^ day. On the 9^th^ day, he developed an ARDS with ventilator associated pneumonia for which he was treated with antibiotics and steroids. After the first unsuccessful extubation, he was finally weaned from invasive ventilator support on the 20^th^ day.

In addition to heart failure he presented multiple organ failure (renal, hepatic, and metabolic). He was treated with one session of extracorporeal haemodialysis because of anuric acute renal failure and metabolic acidosis. He developed hepatic failure with elevated liver enzymes and bilirubin as well as a major drop in the level of prothrombin and factor V. However, hepatic function improved on the 5^th^ day. He also presented rhabdomyolysis with a peak of serum creatine phosphokinase at 15000 UI/L on the 3^rd^ day.

Throughout his stay in ICU, he was sedated with propofol and sufentanil mainly to treat his ARDS. During the weaning of these drugs, he presented agitation, tachypnoea, sweating, and mydriasis, requiring treatment by cyamemazine, buprenorphine, and clorazepate. After a toxicologic analysis of his past drug abuses and the chronology of the therapies we introduced, we diagnosed an opioid-specific withdrawal syndrome.

## 3. Discussion

### 3.1. Pathophysiology

Serotonin is a molecule produced by both chromaffin cells and brain stem cells and plays a key role in the regulation of emotions, pain, muscle tonus, cardiovascular function, temperature, and haemostasis [4]. Moreover, in vitro studies show that the serotonin system is also involved in the pathogenesis of anaesthesia-induced malignant hyperthermia.

Clinical manifestations of serotonin syndrome are caused by overactivation of both central and peripheral serotonin receptors. The ensuing autonomic hyperactivity, neuromuscular abnormalities, and mental-status changes make this syndrome life-threatening. Although the disease is well identified, it can easily be under-diagnosed since the symptoms are not specific and very variable. The Hunter criteria should be used to aid diagnosis [5]. Classically, patients present with akathisia, tremors, clonus, muscular hypertonicity, hyperthermia, high blood pressure, and tachycardia, often associated with biologic disorders such as hyperglycaemia, DIVC, lactic acidosis, and rhabdomyolysis. Many drugs are known to induce a serotonin syndrome [1], including monoamine oxidase inhibitors, tricyclic antidepressants, selective serotonin recapture inhibitors, MDMA abuse, and tramadol. The effects of MDMA on the cardiovascular system are probably due to increased noradrenaline blood levels and include hypertension, damage to blood vessel walls, intravascular thrombosis, cerebral infarcts, tachycardia, hypotension, and cardiac dysrhythmias [6, 7].

This is the first case describing cardiac arrest caused by MDMA-induced serotonin syndrome with full recovery after extra-corporeal life support [8]. In our case, the patient presented cardiac arrest following ventricular tachycardia and fibrillation. The exams we ran afterward revealed no signs of cardiopathy anterior to the electrical storm and there were no predictive factors such as renal or left ventricular insufficiency [9]. The mechanisms of MDMA cardiac toxicity found in vitro include oxidative stress caused by its metabolites, catecholaminergic stimulation, mitochondrial dysfunction, and maybe coronary vasospasm. In the present case, arrhythmia may be due to a high blood level of serotonin or to the MDMA intoxication itself [6, 7]. Our patient also developed acute kidney failure, treated with extracorporeal renal epuration. Serotonin syndrome related renal failure is well described in literature and its causes have been identified [10]. In the present case, several factors explain the acute renal failure, including acute circulatory failure with cardiac arrest, serotonin intoxication, and rhabdomyolysis despite fluid therapy.

### 3.2. Management of MDMA Overdose

The management of MDMA toxicity includes four major points: management of body temperature, use of a serotonin antagonist, control of autonomic instability, and discontinuance of all serotoninergic drugs.

In the present case, we believe that ECLS was the best treatment to restore hemodynamic stability, to treat potential refractory arrhythmia caused by the serotoninergic syndrome, and to manage body temperature. The use of ECMO, already described by Thakkar et al. (2017), appears to be the most important nondrug treatment we initiated. ECLS enabled us to run an effective and quick cooling therapy [11]. We reached the targeted temperature within a few hours, improving prognosis and avoiding the use of antipyretics and of bromocriptine [7]. There is a parallel to be drawn with the way we treated hyperthermia in this case and accidental hypothermia caused by noyade. In addition, multiple organ failure syndrome lasted less than four days, since sustaining blood flow by ECLS prevented its progression.

As expected, the hyperthermia caused by muscle contracture did not respond to dantrolene perfusion [12]. Perfusion of a serotonin antagonist such as cyproheptadine or chlorpromazine [13] could have been discussed to treat the serotonin syndrome and reduce body temperature. However, their effectiveness only appears in case report series and has not yet been satisfactorily proven. Our review of the literature revealed that no work has been done to evaluate if MDMA and its metabolites are removable by dialysis [10, 14]. However, some authors recommend it and, in our case, it may have reduced the intensity of clinical symptoms.

In conclusion, as evoked by Davies [7], the use of ECLS offers several therapeutic advantages in the case of toxic cardiac arrest and serotonin syndrome: hemodynamic support of autonomic instability with body temperature management, treatment of organ failure (liver, renal), and end organ perfusion.
